# The Low Density Matter (LDM) beamline at FERMI: optical layout and first commissioning

**DOI:** 10.1107/S1600577515005743

**Published:** 2015-04-21

**Authors:** Cristian Svetina, Cesare Grazioli, Nicola Mahne, Lorenzo Raimondi, Claudio Fava, Marco Zangrando, Simone Gerusina, Michele Alagia, Lorenzo Avaldi, Giuseppe Cautero, Monica de Simone, Michele Devetta, Michele Di Fraia, Marcel Drabbels, Vitaliy Feyer, Paola Finetti, Raphael Katzy, Antti Kivimäki, Viktor Lyamayev, Tommaso Mazza, Angelica Moise, Thomas Möller, Patrick O’Keeffe, Yevheniy Ovcharenko, Paolo Piseri, Oksana Plekan, Kevin C. Prince, Rudi Sergo, Frank Stienkemeier, Stefano Stranges, Marcello Coreno, Carlo Callegari

**Affiliations:** a Elettra-Sincrotrone Trieste, I-34149 Trieste, Italy; b Graduate School of Nanotechnology, University of Trieste, I-34127 Trieste, Italy; c Department of Chemical and Pharmaceutical Sciences, University of Trieste, 34127 Trieste, Italy; d CNR-IOM TASC, Area Science Park Basovizza, I-34149 Trieste, Italy; e Laboratory of Quantum Optics, University of Nova Gorica, Nova Gorica, Slovenia; f CNR-ISM, Area della Ricerca di Roma 1, I-00015 Monterotondo Scalo, Italy; g Dipartimento di Fisica, Università degli Studi di Milano, Milano, Italy; h Department of Physics, University of Trieste, Trieste, Italy; i EPFL, CH-1015 Lausanne, Switzerland; j Peter Grünberg Institute (PGI-6) and JARA-FIT, Research Center Jülich, 52425 Jülich, Germany; k University of Freiburg, D-79085 Freiburg, Germany; l European XFEL, D-22607 Hamburg, Germany; m TU Berlin, D-10623 Berlin, Germany; n Sapienza Università di Roma, I-00185 Roma, Italy; o CNR-ISM, Area Science Park, I-34149 Trieste, Italy

**Keywords:** beamline, free-electron laser, low-density matter, photon transport, metrology

## Abstract

The Low Density Matter (LDM) beamline has been built as part of the FERMI free-electron laser (FEL) facility to serve the atomic, molecular and cluster physics community. After the commissioning phase, it received the first external users at the end of 2012. The design and characterization of the LDM photon transport system is described, detailing the optical components of the beamline.

## Abstract 2

A description of the LDM beamline of FERMI is given, with a detailed description of the photon transport.

## 1. Introduction   

The Low Density Matter beamline (LDM) is an instrument for experiments involving molecular beams in combination with XUV/soft-X-ray radiation produced by the FERMI FEL; the layout of FERMI and the main properties of its light (high brilliance, short pulse length, variable polarization, coherence) have been described before (Allaria *et al.*, 2010 ▶, 2012 ▶) and are summarized in Table 1 ▶. The 100–4 nm wavelength range is covered by two distinct light sources: the long-wavelength FEL-1 (100–20 nm) and the short-wavelength FEL-2 (20–4 nm). The photon beam paths of the two sources merge in the safety hutch and are transported to the experimental section *via* a common set of optics. The beamline was commissioned in 2012 and is undergoing rapid development. A modular end-station (Lyamayev *et al.*, 2013 ▶) for the production of supersonic beams of atoms, molecules or clusters has been installed. The beamline is now open to external users and the first experimental results have been published (LaForge *et al.*, 2014 ▶; Ovcharenko *et al.*, 2014 ▶; Mazza *et al.*, 2014 ▶; Žitnik *et al.*, 2014 ▶). This paper describes the parts of the LDM beamline not previously reported (Allaria *et al.*, 2010 ▶; Lyamayev *et al.*, 2013 ▶), in the following sections: Photon transport system, Optics characterization, Focusing performance, Beamline transmission and geometrical losses, Commissioning and present status of the beamline.

## 2. Photon transport system   

**Figure 1:**
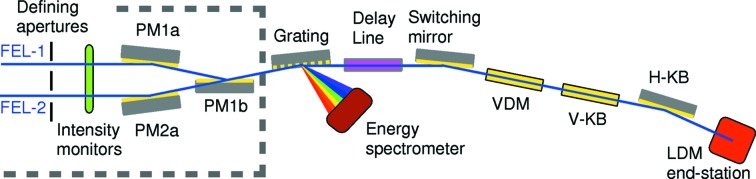
The photon beam transport and diagnostics system of FERMI. The two FEL undulator lines are visible on the left, inside the safety hutch (dashed line). The LDM endstation is in the bottom-right corner. The parameters of the optics are reported in Table 2 ▶.

The photon analysis, delivery and reduction system (PADReS) consists of the section of the machine from the exit of the undulators to the endstations. A set of plane mirrors (PM1a and PM1b serving FEL-1; PM2a serving FEL-2) is installed in the so-called safety hutch. Each FEL source has its own beam diagnostics and beam conditioning instruments, such as the intensity and beam position monitors, beam defining apertures and gas absorber  (Zangrando *et al.*, 2009 ▶). At the end of the safety hutch the two photon beam paths enter the PM1b chamber (see Fig. 1 ▶ ▶), where one or the other can be selected.

Outside the safety hutch, the energy spectrometer PRESTO  (Svetina *et al.*, 2011 ▶) records the spectrum of each pulse by diffracting and detecting 1–2% of the total intensity, while delivering the essentially unperturbed beam downstream. It employs two plane substrates, ruled as gratings only in their central parts (60 mm over a total length of 250 mm). The rulings have a variable line spacing along the longitudinal direction (along the photon beam propagation direction) in order to focus the diffracted radiation onto a movable two-dimensional detector that tracks the focal curve. A computer calculates the one-dimensional spectrum from the image, as well as the central wavelength, bandwidth, horizontal and vertical projection on a shot-by-shot basis, and this information can be stored with the experimental data. The two gratings cover the whole wavelength range of FERMI as provided by FEL-1 (low-energy grating, LE) and FEL-2 (high-energy grating, HE).

A split-and-delay line (AC/DC: AutoCorrelator/Delay Creator) is installed after the spectrometer, for pump and probe experiments. It is based on the splitting and subsequent recombination of the incoming wavefront after passing through two different branches (one of variable length, the other of fixed length), by means of eight Au-coated plane mirrors, four for each path. In the variable length branch, two mirrors move on 900 mm-long linear guides with an accuracy of 10 µm, as measured by an optical encoder. Changing the positions of these two mirrors produces a difference of the two path lengths and introduces a time delay variable between −1.5 ps and 30 ps in steps of 0.3 fs. The mirrors of the two branches operate with two different grazing incidence angles: 2° in the fixed branch, 3° in the variable branch. This difference results in different transmission coefficients of the light, which have been calculated considering perfect mirrors (flat and free of contamination). For the fixed-length branch the transmission of the four mirrors, in the wavelength range from 4 to 100 nm, is about 80% for *s* polarized radiation and about 50–65% for *p* polarization. The transmission of the other branch is about 70% for *s* polarization and about 30–50% for *p* polarization. For selected wavelength ranges the length of each branch can be further extended by inserting four more multilayer (ML) mirrors operating at 45° incidence, thus introducing an additional delay variable between 0.3 ns and 1.3 ns. The type of multilayers must be chosen according to the experimental needs; we note that, even at their design wavelength, four additional ML mirrors reduce considerably the light transmission. Seven out of eight mirrors have motorized pitch-and-roll movements, providing fine control of the alignment and the attainment of very good spatial overlap of the half beams in the experimental stations. Overlap is visually evaluated by inspecting the two beams on a YAG screen at the centre of the end-station, and later optimized by maximizing a suitable experimental signal. It is possible to filter the radiation independently in the two branches of the AC/DC unit: before the recombination mirror, an easily accessible section that can host three filters for each branch gives users the possibility of mounting filters suited to their experimental needs. To install filters from air into vacuum requires a downtime of about 12 h.

The three-way switching mirror chamber selects which beamline is in use. The plane switching mirror (SW) serving LDM deflects the beam in the horizontal direction, and has a dual coating (graphite and iridium, each covering half the width of the mirror), to maximize the reflectivity for FEL-1 and FEL-2, respectively. Downstream of the switching mirror, the beam undergoes three further reflections: from a vertical deflecting mirror (VD) and from two Kirkpatrick–Baez (K–B) mirrors.

The K–B system consists of two thin plane mirrors clamped at their sides and bent *via* two mechanical pushers acting independently on their respective clamps in order to attain the best elliptical profile as shown in Fig.  5 of Raimondi *et al.* (2013 ▶). The K–B configuration (Kirkpatrick & Baez, 1948 ▶) decouples the horizontal and vertical focusing. The active shape allows users to finely adjust the focal length, making provision for the fact that the position of the last undulator (*i.e.* the nominal source points) of FEL-1 and FEL-2 differ by ∼7 m. Likewise, the K–B active optics can compensate and correct astigmatism and defocusing effects originating from non-ideal profiles of the preceding plane mirrors, that are discussed in the next section.

### 2.1. Optics characterization   

All the optics described above were characterized in the Elettra metrology laboratory by means of atomic force microscopy (AFM) and white-light interferometry, to cover the spatial high-frequency range (10 µm to 1 mm) and determine the roughness. The r.m.s. roughness has been measured to be below 2 Å, small enough to consider the surface scattering negligible, as verified during the commissioning of the beamline. The spatial low frequencies (from 0.5 mm to the length of the mirror) of the optical surfaces, *i.e.* the slopes and figures, have been measured with a long trace profiler (LTP) (Rommeveaux *et al.*, 2008 ▶). All optics meet specifications and a summary of the results of the metrological inspection (residual radius of curvature, peak-to-valley and slope error r.m.s.) for the plane mirrors is reported in Table 3 ▶.

The mechanical bending system of the two K–B mirrors has also been tested and optimized using the *Adaptive Correction Tool* software (*ACT*) (Signorato *et al.*, 1999 ▶). The best profiles, *i.e.* those closest to the nominal elliptical form, have been achieved (Raimondi *et al.*, 2013 ▶); their sagittas (distance from the centre of the arc formed by the mirror to the base of the arc) have been measured to be ∼230 µm for the horizontal focusing mirror and ∼160 µm for the vertical focusing mirror. The presence of residual radii of curvature, slope errors and figure errors may cause a variation of the actual focal distance and intensity distribution of the virtual source seen by the K–B focusing system that is discussed in the next section.

### 2.2. Focusing performance   

Calculations of the expected spot size and shape have been carried out for both sources (FEL-1 and FEL-2) over their whole wavelength range, using the codes *SHADOW* (Sanchez del Rio *et al.*, 2011 ▶), based on ray tracing, and *WISE* (Raimondi *et al.*, 2015 ▶), based on physical optics. The sources have been modelled as Gaussian beams, with the measured values of the FERMI radiation (source size, divergence, beam propagation factor *M* ^2^) as input parameters. The beams were propagated along PADReS (both ‘ideal’ and ‘as measured’ mirrors are considered); there is good agreement between the physical-optics and the ray-tracing approach except for some diffraction effects absent in the latter simulations.

**Figure 2:**
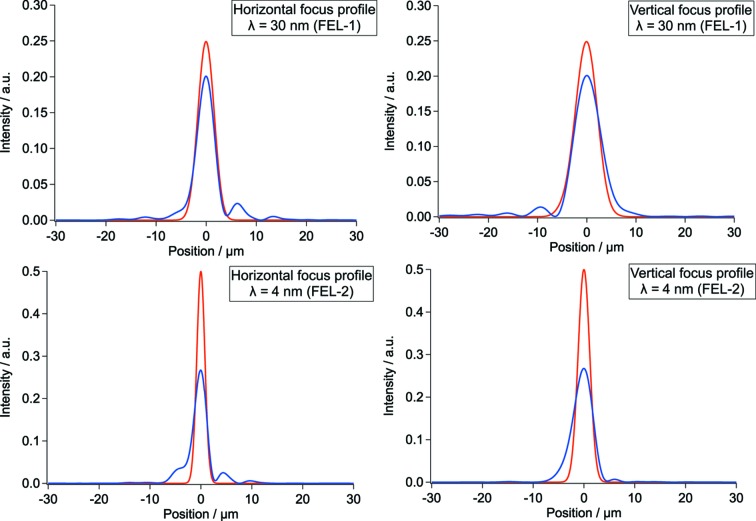
Simulated focal spots for the LDM beamline in the case of ideal (red) and real (blue) mirrors; the *WISE* program was used. The intensity profiles are calculated for FEL-1 at 30 nm and FEL-2 at 4 nm, and are displayed along the vertical and the horizontal directions. The diffraction effect is due to the finite size of the mirrors. The smallest achievable spot sizes (FWHM) are predicted to be ∼4 µm × 6 µm for FEL-1 and ∼3 µm × 5 µm for FEL-2. The areas are all normalized to unity in order to compare the size of the spot irrespective of the intensity of the incident beam.

Calculated spot profiles in the case of smallest spot size (4 µm × 6 µm FWHM for FEL-1 and 3 µm × 5 µm FWHM for FEL-2) are shown in Fig. 2 ▶. Here the effect of the non-ideal mirror shape can be seen as a broadening of the spots and the appearance of some diffraction peaks. The unavoidable diffraction effect is due to the finite size of the optical elements and becomes smaller as the wavelength decreases; in any case the beam quality remains high, as experimentally confirmed during the commissioning phase.

We emphasize the great importance of the bendable K–B system in the focusing section. Besides the need to accommodate, as already mentioned, different source positions for FEL-1 and FEL-2, deviations of the transport optics from a perfect plane profile (slope errors) cause a variation of the distance and intensity distribution of the virtual source as seen by the K–B mirrors. As an example, at 32.5 nm (FEL-1) we estimate that the virtual source becomes astigmatic with the horizontal and vertical waists located about 9.70 m and 2.94 m, respectively, downstream of the nominal position. This effect can be easily handled and compensated by properly changing the curvature of the K–B mirrors.

To conclude this section, we mention some techniques we adopt to optimize focusing of the photon beam according to the needs of the user. For less demanding experiments that tolerate a larger spot size (above ∼20 µm FWHM) in exchange for easier and faster operation, we use a simple fluorescent screen (YAG, YAP or phosphor) inserted at the nominal focal plane; we have also investigated the use of multi-photon ionization of rare gas atoms as a quick feedback signal (see §3).

For studies requiring micro-focusing, the most informative diagnostic is a wavefront sensor (WFS) of the Hartmann type (Mercère *et al.*, 2003 ▶). The WFS is based on an array of 13 mm × 13 mm pin-holes coupled to a CCD camera (1024 × 1024 pixels; pixel size 24 µm × 24 µm); the instrument allows the user to measure the wavefront at the location of the array (in our case, 1.2 m downstream of the focal point, *i.e.* of the FEL–sample interaction region); specifically, the wavefront sensor software calculates the intensity distribution of the beam (typically a mix between several modes resulting in a *noisy hyper-Gaussian* intensity profile) and the wavefront deviation (residual) from the ideal propagation shape. For a reasonably smooth wavefront, dedicated software back-propagates the result of the measurement to the focal point and reconstructs the focal spot.

**Figure 3:**
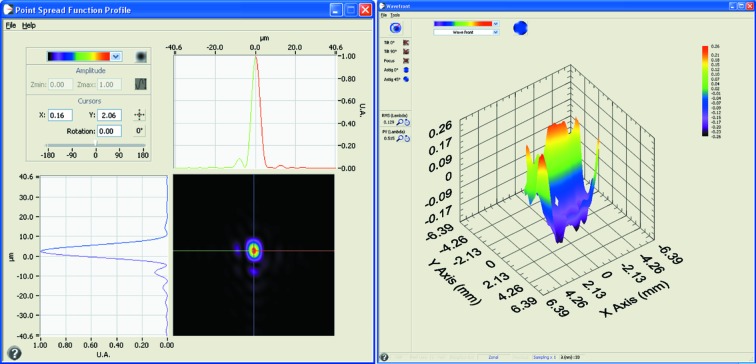
The left-hand panel shows the best focal spot of 5 µm × 8 µm obtained during the wavefront sensor measurement campaign. This spot is reconstructed *via* software from the wavefront measured 1 m out of focus behind the LDM end-station. The right-hand panel shows the wavefront residuals (after tilt compensation and subtraction of the ideal propagation wavefront).

We performed a measurement campaign on the LDM end-station to ascertain the influence of K–B mirror bending on spot size, and consequently refine the mirror shape, as well as to confirm the accuracy of the WISE simulations. The wavefront residuals allow an excellent optimization of the focal spot: the strategy is to flatten the wavefront by bending the mirror and finely adjusting the system angles (*i.e.* pitch and roll of K–B mirrors, and incidence angles of the light). In particular, in order to optimize the mirror curvature we tried to minimize the aberrations that were quantified in terms of Zernike coefficients. The Hartmann sensor software is able to compute these coefficients; consequently it allows the operator to understand how to adjust the pusher motors in order to reduce the aberrations. It is indeed very easy to correct the optical aberrations and reach the best wavefront profile (thus, focal spot) that this mechanical system can attain. Fig. 3 ▶ shows a single-shot image of the best focal spot achieved, at a FEL wavelength of 30 nm, with this technique: 5 µm × 8 µm (FWHM). In this case we obtain a wavefront residual r.m.s. of 9 nm. Simulations obtained with the *WISE* code, and based on the ideal elastic deformation of the mirrors, produce a focal spot of 4 µm × 6 µm (FWHM); we conclude that with the wavefront sensor as a feedback it is possible to bend the optics to perform very close to the ideal limit of the mechanical system.

### 2.3. Beamline transmission and geometrical losses   

**Figure 4:**
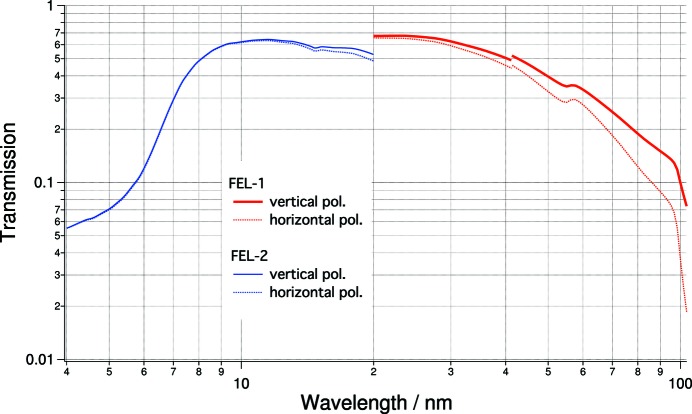
Calculated transmission of the LDM beamline optics for the two FEL sources and the two linear polarizations (FEL-1: red traces; FEL-2: blue traces; vertical polarization: solid traces; horizontal polarization: dotted traces). The mirrors delivering the photon beam to the LDM end-station are PM1a, PM1b, LE grating for FEL-1; PM2a and HE grating for FEL-2; mirrors SW, VD, H-KB, V-KB are common to both. The geometrical losses have been included in the calculation. The discontinuity at 41.3 nm is due to the use of two different databases: Palik (1997 ▶) and Henke *et al.* (1993 ▶).

Overall, in the basic configuration (delay line withdrawn) seven mirrors are used for FEL-1 and six for FEL-2. Using the parameters reported in Table 2 ▶ as input for the computer codes *REFLEC* (Schafers & Krumrey, 1996 ▶) and *IMD* (Windt, 1998 ▶), we calculated the reflectivity of each mirror for horizontal/vertical polarizations. The calculated overall transmission of the beamline is shown in Fig. 4 ▶.

Due to the beamline geometry (five mirrors reflecting in the horizontal plane, two in the vertical plane for FEL-1; four and two for FEL-2), the vertical and horizontal polarizations are not equally transmitted to the endstation, thus the ellipticity varies across the whole wavelength range. The linear horizontally polarized light suffers greater losses than the vertically polarized one. While this effect is negligible below 40 nm, above this wavelength it rapidly increases. The choice of APPLE-2 undulators for FERMI (Allaria *et al.*, 2014 ▶) allows smooth control of the polarization of the emitted radiation: *i.e.* one can set the FEL polarization to be elliptical upstream of the transport optics, in order to have circularly polarized radiation at the end-station (Allaria *et al.*, 2012 ▶, 2014 ▶).

The beam divergence in the far-field, 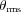, is proportional to the wavelength λ (Table 1 ▶): as a consequence there are geometrical losses due to the finite size of the mirrors that can affect the overall transmission. These geometrical losses are higher at longer wavelengths and become negligible at shorter wavelengths. The geometrical acceptances at different wavelengths have been determined using ray-tracing simulations, and are reported in Table 4 ▶.

## 3. Commissioning and present status of the beamline   

During the beamline construction period, commissioning measurements were performed with a prototype end-station equipped with a velocity map imaging/ion time-of-flight spectrometer (VMI/TOF). The original VMI spectrometer and its further improvements have been described previously (O’Keeffe *et al.*, 2012 ▶).

**Figure 5:**
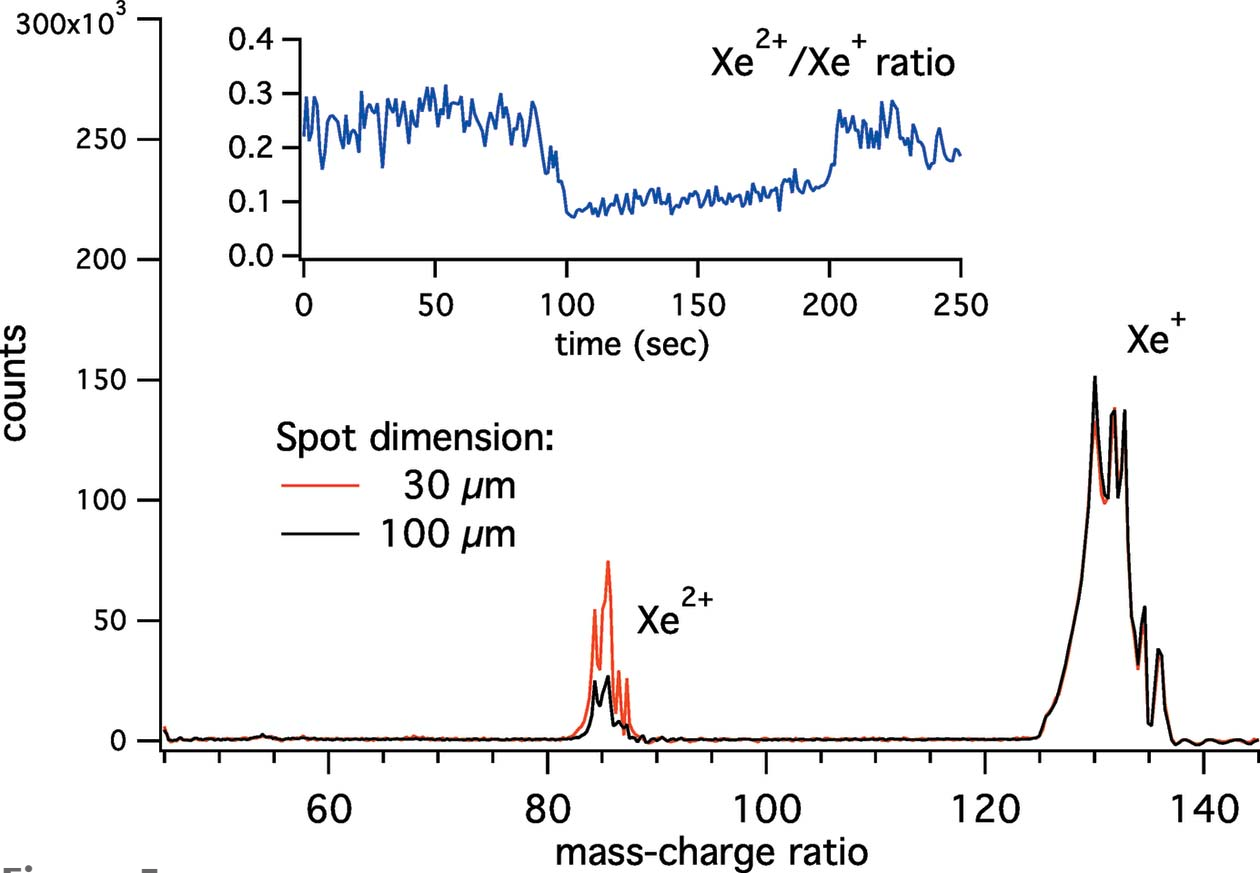
Ion time-of-flight (TOF) mass/charge spectra of Xe taken at λ = 52.22 nm (23.74 eV) for different focusing conditions. Each spectrum is a sum over several spectra; all spectra have been recorded with the same FEL intensity. In the inset (blue line) we show the Xe^2+^/Xe^+^ intensity ratio as a function of time while changing the curvature of the K–B mirrors.

One of the test measurements is shown in Fig. 5 ▶. At the time of the commissioning the FEL pulse length was 120 fs and the energy per pulse was 60 µJ. In order to test the capabilities of the adjustable focusing system we measured ion TOF spectra of Xe atoms exposed to FEL pulses, observing charge states indicative of multi-photon absorption; the intensity ratio of different charge states as a function of time while changing the bending of the K–B mirrors (inset to Fig. 5 ▶) is a good indicator of the reproducibility of the focusing system for small curvature changes. While highly charged states of Xe obtained upon FEL irradiation have been reported in the literature (Sorokin *et al.*, 2007 ▶) for photon energies within the 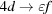 giant resonance (93 eV), to our knowledge only sequential double ionization of Xe has been reported at 23.0 and 24.3 eV (Mondal *et al.*, 2013 ▶), at average intensities of 2–3 × 10^13^ W cm^−2^.

The LDM beamline now features a modular end-station accommodating a broad range of detectors and systems for producing targets. The combined capabilities of the photon source (high brilliance, short pulse length, variable polarization, coherence), photon transport (variable-focusing optics) and endstation allow the investigation of many targets, such as very dilute systems, matter under extreme irradiation conditions (multiple electronic excitation, multiple ionization, Coulomb explosion, non-linear optics) and dichroism. The split-and-delay line described above, as well as a synchronized optical laser (Cinquegrana *et al.*, 2014 ▶), allow time-resolved experiments with different combinations of femtosecond pulses.

## Figures and Tables

**Table 1:** FEL-1 and FEL-2 parameters. [see also Giannessi *etal.* (2012 ▶) and Allaria *etal.* (2015 ▶)]

	Value		
Parameter	FEL-1	FEL-2	Units
Wavelength†	10020	204	nm
Pulse length (FWHM)‡	30100	100	fs
Bandwidth (FWHM)§	1 10^3^	1 10^3^	
Polarization	Variable	Variable	
Repetition rate	10§; 50†	10§; 50†	Hz
Energy per pulse§	>50	>10	J
Divergence (r.m.s.)¶	1.25	1.5	rad

† Design.

‡ Calculated.

§ Achieved.

¶ in nm.

**Table 2:** Parameters of the optics *d*: distance from the nominal source points (FEL-1/FEL-2); *w*:width; *l*:length; :grazing incidence angle. PM2a and SW have two coatings, each covering half the width of the mirror, and their position can be adjusted sideways to use one or the other coating. Orientation (H, V) refers to the deflection plane.

Mirror	*d* (m)	*w* *l* (mm)	()	Coating	Shape (orientation)
PM1a	48.1/	20 400	2.5	Graphite	Plane (H)
PM1b	54.3/	20 250	5	Graphite	plane (H)
PM2a	/41.4	20 300	2.5	Graphite/Au	Plane (H)
PRESTO-LE	57.5/49.8	20 250	2.5	Graphite	VLS plane grating (H)
PRESTO-HE	57.5/49.8	20 250	2.5	Au	VLS plane grating (H)
SW	77.5/69.9	25 480	2	Graphite/Ir	Plane (H)
VD	90.0/82.3	20 390	2	Au	Plane (V)
H-KB	95.6/87.9	40 400	2	Au	Active (V)
V-KB	96.1/88.5	40 400	2	Au	Active (H)

**Table 3:** Measured optical parameters *R*: radius of curvature; Res PtV: residual peak-to-valley displacement after best-sphere subtraction; Res slope error: residual slope error (r.m.s.) after best-sphere subtraction.

Mirror	*R* (km)	Res PtV (nm)	Res slope error (rad)
PM1a	42.3	120	0.89
PM1b	10.6	46.5	0.56
PM2a	28.1	135	0.95
PRESTO-LE	29.7	36	0.44
PRESTO-HE	27.1	22	0.45
SW	34	78.5	0.59
VD	154.6	42.3	0.32

**Table 4:** Geometrical acceptance (due to the finite mirror sizes and the photon beam divergence), reflectivity and overall transmission of the photon beam transport system

Wavelength (nm)	FEL	_rms_ (rad)	Geometrical acceptance (%)	Reflectivity (%)	Overall transmission (%)
65	1	81.3	41.3	54.8	22.7
52	1	65	54.5	58.0	31.6
43	1	53.8	68.1	57.4	38.8
32	1	40	86.9	59.8	51.3
20	1	25	99.3	56.7	56.6
20	2	30	97.0	54.6	55.3
10	2	15	>99	64.5	65.0
8	2	12	>99	54.8	54.3
6	2	9	>99	12.0	11.9
4	2	6	>99	5.0	4.98
